# Secretome profile selection of optimal IVF embryos by matrix-assisted laser desorption ionization time-of-flight mass spectrometry

**DOI:** 10.1007/s10815-019-01444-7

**Published:** 2019-05-15

**Authors:** Ray K. Iles, Fady I. Sharara, Raminta Zmuidinaite, Galal Abdo, Sholeh Keshavarz, Stephen A. Butler

**Affiliations:** 1 MAP Sciences Ltd., Priory Business Park, Bedford I-lab Stannard Way, Bedford, MK44 3RZ UK; 2 Virginia Center for Reproductive Medicine, Reston, VA USA; 3 George Washington University, Washington, DC USA

**Keywords:** Blastocyst, Secretome, Proteomics, Mass spectrometry, Non-invasive, Culture media

## Abstract

**Purpose:**

Selecting an embryo at the transfer stage with the best chance of a successful pregnancy is still largely dependent on preceding subjective evaluation of morphokinetics. Expensive prenatal genomic profiling has been so far proved ineffective. Proteomics and metabolomics are promising new approaches to assess embryo viability, but methodologies are often complex and do not lend themselves to rapid analysis in the critical time between blastocyst formation and embryo transfer. Here, we used matrix-assisted laser desorption ionization time-of-flight (MALDI ToF) mass spectrometry to assess the secretome of blastocysts in the minutes prior to embryo transfer and correlated spectral features with pregnancy outcome.

**Methods:**

Four hundred one samples of spent blastocyst culture media were collected from embryo cultures at the time of embryo transfer, of which 136 were used to construct the predictive model. The media samples were frozen at − 20 °C and stored for analysis. Sample analysis was conducted in batches using 1 μl of spent embryo in direct MALDI ToF mass spectral analysis. Quantitative characteristics within this mass range (2000–17,000 *m*/*z*) were used to generate a score for selected mass regions (bins) in order to predict pregnancy outcome for each sample.

**Results:**

With a simple algorithm based on nine mass bins within the 2000–10,000 *m*/*z* region, it was possible to identify samples with the best chance of becoming an ongoing pregnancy (positive predictive value of 82.9%, *p* = 0.0018).

**Conclusion:**

A simple, direct and rapid analysis of spent culture fluid from blastocysts at the point of embryo transfer can quickly identify optimal embryos with the best chance of achieving ongoing pregnancy. Methods like this, which take less than 20 min to perform, could dramatically improve the approach to embryo selection and live births.

## Introduction

Selecting an embryo at the transfer stage with the best chance of a successful pregnancy is still largely dependent on preceding subjective evaluation of morphokinetics, either visually or by time-lapse camera [1]. Other approaches require a more invasive method to perform molecular analysis of genes or chromosomes to eliminate embryos with a greater risk of miscarriage, but this requires a procedure involving embryo biopsy. Both non-invasive and invasive approaches are now commonplace, but despite the widespread application of these methods, there has been little improvement in pregnancy rates or live birth rates from assisted reproduction in recent years. The latest data from the European IVF-Monitoring Consortium indicates that there has been no significant improvement in pregnancy rates or birth rates from assisted reproduction in the last 3 years studied [2]. Recently, preimplantation genetic screening (PGS) has been shown not to have increased live birth rates [3] and it has been questioned why such testing was introduced as it is invasive, expensive and resulted in changes in clinical practice (i.e. freezing of embryos) in order to give time for results to be obtained prior to embryo transfer [4–6].

Several recently published reviews describe the latest developments in non-invasive approaches to assess embryo viability and elegantly evaluate the extensive literature on morphokinetics [1], proteomics and metabolomics [1, 7, 8], and small non-coding RNA [8]. The RNA approaches are still in their infancy, offering mixed results [8], and morphokinetics, after 30 years of implementation, is yet to convincingly correlate with an increase in live birth rates. In contrast, metabolomic and proteomic approaches are emerging as promising non-invasive procedures to evaluate embryo viability [7].

Advances in mass spectrometry have meant that the metabolomic and proteomic approaches are now very sensitive and powerful. It is assumed that embryos resulting in successful pregnancy differ in their metabolic, proteomic, and secretomic profiles compared with embryos that do not. Only a few studies are ongoing to identify a molecular signature that can be detected by non-invasive evaluation of the embryo culture medium [9–11]. Others have identified key proteins produced by embryos, using similar approaches, which offer an insight into the possibility of media supplementation to improve optimal maturation and subsequently improve implantation rates [12, 13]. However, this does not directly address the clinical need of best embryo selection. The most promising studies on embryo selection, to date, involve methods which combine both proteomics and metabolomics with the more established time-lapse assessment [14, 15].

One group in particular has approached the clinical need directly using electrospray ionization mass spectrometry (ESI MS) to identify unique protein signatures in positive and negative outcome embryos [16]. In a similar method to that described here, the group has adopted mass spectrometry fingerprinting rather than identifying specific target molecules as the clinical marker. However, they differ in their technical approach by using electrospray ionization (ESI) and hence are limited to low mass range spectral fingerprints [17]. In combination with complex mathematical modeling, it is possible to use these spectral signatures to fit data into one of two groups (outcome positive and outcome negative). A proposed mathematical model would then select the most appropriate embryo based on that fitted data. However, while these methods are very exciting from a research perspective, the complexity of the sample preparation, preprocessing, cost, and technical skill of the operator mean that tests like these are not likely to emerge as methods that will work in the clinical setting.

In contrast, we have previously demonstrated that it is possible to quickly analyze culture media from spent blastocysts using matrix-assisted laser desorption ionization time-of-flight mass spectrometry (MALDI ToF MS) [18]. This is technically a much simpler and more direct method of mass spectrometry than ESI. Furthermore, by examining higher mass regions and hence important protein cytokines, divided into mass bins, rather than individual ions, it has been possible to develop simple risk algorithms for prenatal diagnosis following the MALDI ToF MS analysis of early pregnancy urine [19]. Using a combination of these two earlier approaches [18, 19], we set out to investigate the feasibility of analyzing culture media using MALDI ToF MS spectral fingerprints immediately prior to embryo transfer to establish a clinically practical approach to selecting the embryo with the best chance of establishing an ongoing pregnancy.

## Materials and methods

### Patients and samples

Patients recruited to the study included women undergoing routine-assisted reproduction cycles at the Virginia Center for Reproductive Medicine (VCRM), IVF clinic, between March 2014 and August 2016. All couples gave consent for the culture media to be used. The study was approved by VCRM’s Institutional review board.

**Fig. 1:**
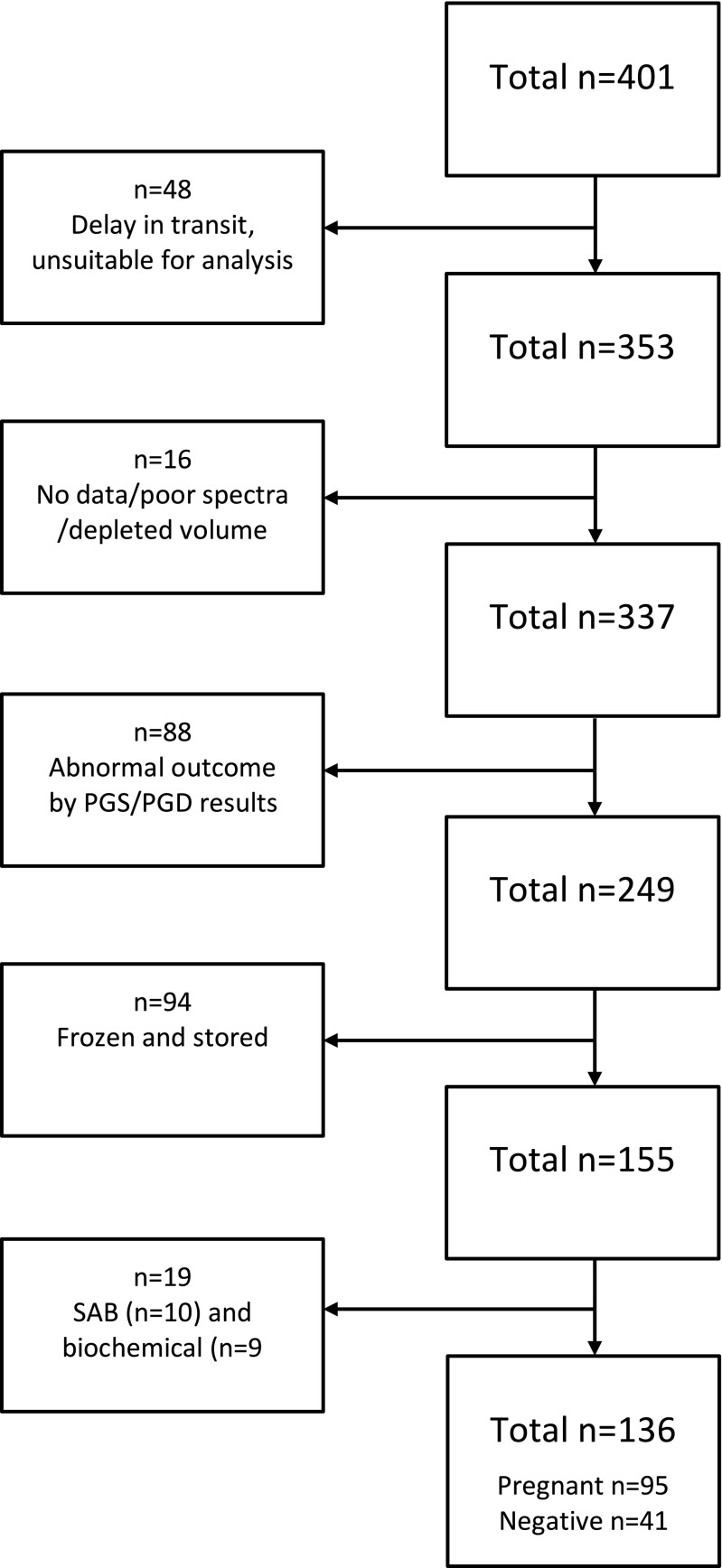
STARD diagram to report flow of samples through the study. Flow diagram illustrating the sample selection process, inclusion and exclusion criteria, and outcome data. Total samples received to date from 11 batches totaling 401 samples. A final 136 samples, comprising 95 with positive pregnancy outcome and 41 negative pregnancy outcome, were analyzed and presented in this study

A total of 401 spent blastocyst culture media samples were collected from 216 women undergoing routine IVF/intracytoplasmic sperm injection (ICSI) cycles. A STARD diagram showing sample processing, inclusion, and exclusion criteria is described in Fig. 1. All women underwent ovarian stimulation using either a long luteal GnRHa or a GnRH antagonist-based protocol, as previously described [20]. All cycles used a mixed protocol using urinary or recombinant FSH (Bravelle, Ferring; GONAL-f, EMD Serono; and Follistim, Merck) and HMG (MENOPUR, Ferring). When at least three follicles reached 16–18 mm in diameter, 5000–10,000 u of urinary HCG (Novarel, Ferring) was administered subcutaneously, and oocyte retrieval was performed 35 h later. Oocytes were rinsed and denuded using a hyaluronidase solution combined with mechanical stripping, and ICSI was performed 4–6 h later. Embryos were cultured in groups under mineral oil in droplets of culture media (Global Medium, Life Global, CT, USA) with 10% serum substitute supplement. All embryos were cultured under 37 °C in a 5% O_2_, 5% CO_2_ environment for 5 days. All embryo transfers were performed on day 5 or 6 post-fertilization. Single embryos were transferred into 50 μl drops of CSC media (Irvine Scientific, CA, USA) for 30 min prior to embryo transfer. The spent media remaining after the embryo transfer, or the 50 μl drop from the non-transferred embryos, represented the total sample used for the analysis in this study. The media was collected, stored in Eppendorf tubes and frozen at − 20 °C prior to being shipped frozen to the research laboratory in the UK for MALDI ToF MS analysis. All embryo transfers were performed under ultrasound guidance using a Wallace SureView catheter (Irvine Scientific, CA, USA) by a single physician (FIS). Of the embryos transferred, 83 were single-embryo transfer (SET–61%), 52 were two-embryo transfer (DET—38%), and 1 was a three-embryo transfer procedure (TET—0.7%). A pregnancy test was performed 10–12 days after embryo transfer. Pregnant patients underwent a vaginal ultrasound at 6–7 weeks, and the number of gestational sacs was recorded in order to confirm pregnancies. Biochemical pregnancies were recorded as negative implantation/pregnancy event.

### Sample preparation

Samples were thawed completely, and an aliquot was pretreated with 2 M dithiothreitol (DTT) solution (1:1) for 30 min and subsequently plated onto a 384-well stainless steel plate compatible with the Shimadzu AXIMA MALDI ToF platform. The plate was prepared with 1-μl layer of sinapinic acid (SA) matrix (20 mg/ml in a 1:2 solution of 0.1% trifluoroacetic acid (TFA) in ddH2O and acetonitrile (ACN)) which was left to air dry. Subsequently, 1 μl of embryo culture fluid sample/DTT mixture was plated on top of the crystalized matrix layer and sandwiched between further layers of 1 μl SA matrix added on top of the spot. One sample per spot was prepared. All spots were left to completely dry and crystalized whereupon the plate was loaded into the MALDI ToF mass spectrometer.

### MALDI ToF MS analysis

The prepared and plated samples were analyzed using MALDI ToF (Shimadzu, AXIMA CFR plus) mass spectrometer which was calibrated using equine cytochrome C (12,362 Da) and/or apomyoglobin (16,952 Da) (ProteoMass, Sigma-Aldrich) for both singly and doubly charged ions. Sample ionization was by a pulsing (50 Hz) nitrogen laser (*λ*max = 337 nm) and mass analysis by time of flight, in linear mode, over a 1.2-m flight tube. For each sample, 500 profiles with 10 laser shots per profile were acquired in a 1000- to 100000-*m*/*z* range, averaged and saved as comma delimited ASCII files.

### Data extraction and analysis

The mass range initially examined using Shimadzu Biotech Launchpad 2.8 software was 1000 to 100,000 *m*/*z* but was ultimately limited to collecting data between 1000 and 27,000 *m*/*z* to reduce files to manageable sizes. The comma delimited ASCII data files were exported to mMASS™ (open-source mass spectrometry analysis software) was subjected to cropping and baseline correction, adjusting precision and relative offset for each sample individually. For each sample, only data corresponding to the region 2000 to 17,000 *m*/*z* was utilized for analysis.

Analysis was carried out using Excel (Microsoft): summed data from 150 bins (each bin of 100 *m*/*z*) from the mass region including 2000 to 17,000 *m*/*z* constituted the total ion signal of the spectra. The resultant 150 bins were each expressed as a percentage of the total spectra ion count corresponding to the area under the curve for our selected region (2000 to 17,000 *m*/*z*). This normalization rendered all spectra comparable in terms of peak intensity as well as corresponding peak mass. Exploratory data analysis of each of the 150 bins was carried out in Excel to determine difference in the spread of values between our two outcomes and identify the bins with the highest positive predictive values for use in the algorithm. Descriptive statistics for each selected bin was calculated and displayed as box and whisker plots using R™—a language and environment for statistical computing [21]. Statistical comparisons, such as Mann–Whitney *U* tests, were performed using StatsDirect™ software (Altrincham, Cheshire, UK).

## Results

Embryo sample outcomes were classified into two groups as either successful, ongoing, positive pregnancy (*n* = 95) or never implanting, negative pregnancies (*n* = 41) and are referred to in this study as “pregnant” or “negative,” respectively. Samples from embryos which resulted in biochemical pregnancies (*n* = 9) or spontaneous abortions (*n* = 10) were excluded (see Fig. 1). Of the positive ongoing pregnancies, 21 were twins; 1 was a triplet, and the remainder (*n* = 73) were singleton.

**Fig. 2:**
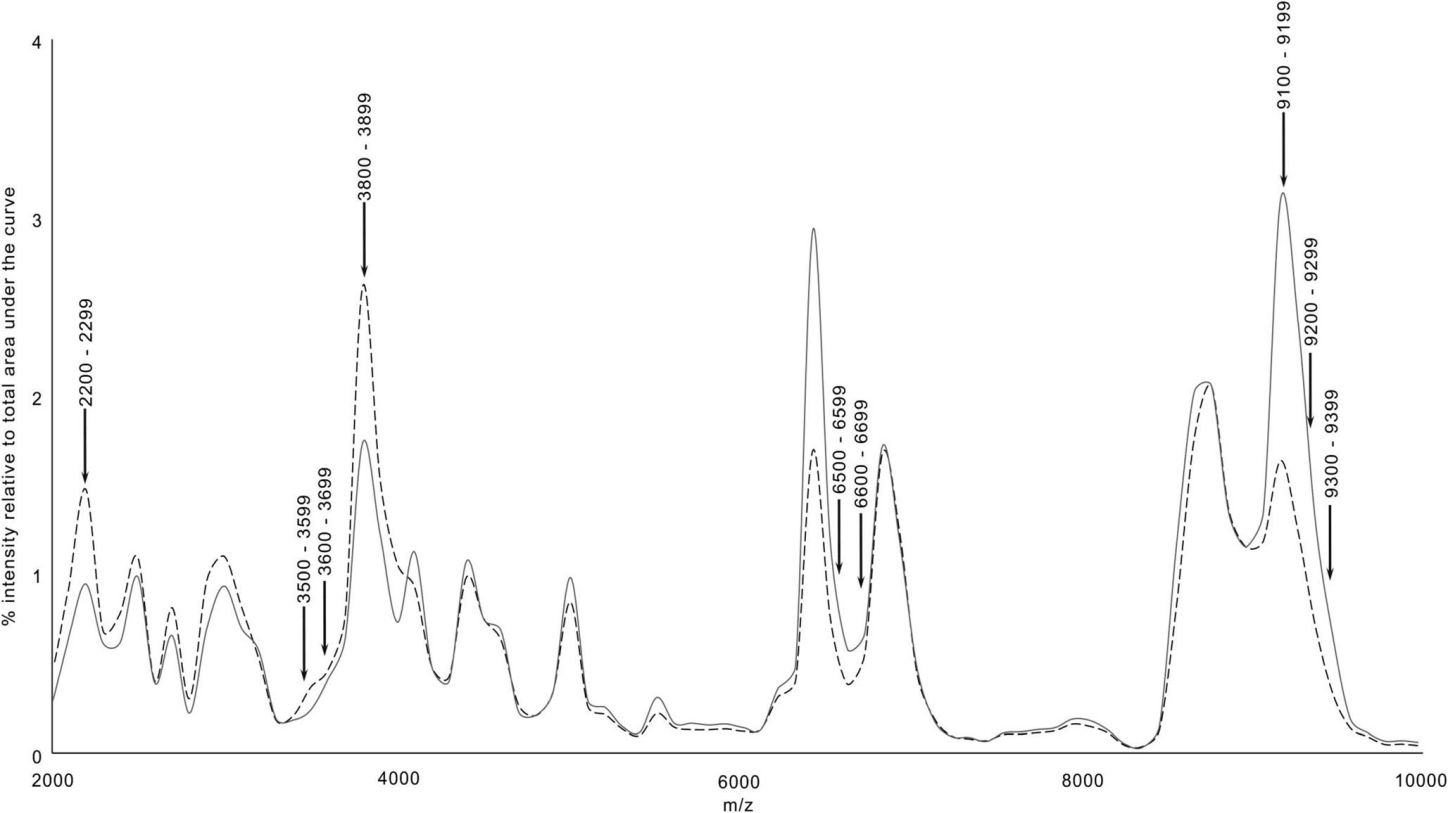
Comparative MALDI spectra of the analysis of media from blastocyst samples resulting in ongoing pregnancies and negative pregnancies. Median values spectra for fresh blastocyst samples taken prior to embryo transfer, illustrating spectral differences between those with a positive pregnancy outcome (pregnant) and those with a negative pregnancy outcome (negative). Spectral differences are quantified at nine specific regions corresponding to 100 *m*/*z* which we term “bins.” Each of the nine bins are indicated by their mass ranges and an arrow pointing at the respective position on the spectral plot over the mass range of 2000–10,000 *m*/*z*. In each case, visible differences can be seen between the median values for samples which went on to viable pregnancies and those that did not

Each sample was analyzed independently using MALDI ToF MS analysis of spent media from blastocyst culture immediately prior to embryo transfer. Analysis gave rise to a unique secretome spectra for each embryo (individual data not shown), and data was collected for all 136 samples eventually included in the study. Specifically, spectral data was acquired from the 2000- to 17,000-*m*/*z* range for each sample and then divided into 150 “bins” each representing a 100 *m*/*z*. Median values for each bin were expressed as percentage intensity relative to the total area under the curve for the entire range. This was performed for all pregnant outcome samples and all negative outcome samples and is shown as an overlaid spectrum in Fig. 2. The algorithm which was eventually designed used regions in the 2000- to 10,000-*m*/*z* range, so for this reason, only this range is shown in Fig. 2. Median values for each of the 150 bins were plotted, and clearly, visible spectral differences could be seen at multiple mass (*m*/*z*) regions for both outcomes (Fig. 2). Each 100-*m*/*z* bin was regarded as an independent variable and processed according to outcome differences.

**Fig. 3:**
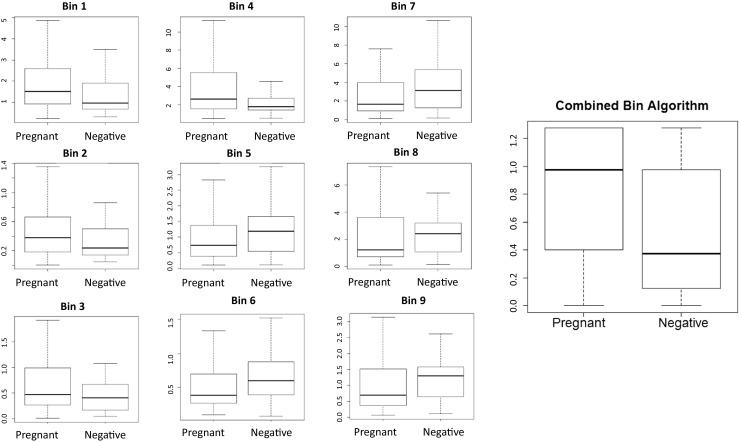
Box and whisker plots for each of the nine selected bins used to construct the test algorithm and box and whisker plot for the combined algorithm. Median values, 75th and 25th centiles, and total range are shown. Mass ranges, cutoffs, and other descriptive statistics for each of the bins are detailed in Table 1. Panel 10 includes the box and whisker plot for the combined algorithm utilizing data taken from all nine bins described in the figure above

The probability/relative risk of having a positive outcome was generated for different cutoff values for each of the 150 bins. Numerous predictive algorithms can be generated by summing the various relative risk (RR) scores from combinations of 100-*m*/*z* bins. These are then tested to provide a modeled spectral scoring algorithm based around a cutoff that provides the best positive predictive value for an embryo that will implant and produce a pregnancy (Fig. 3, panel 10 shown as combined bin algorithm). Thus, any combination of bins can be modeled but only those which can separate outcomes with a high degree of accuracy and good positive predictive value (PPV) were included. Nine such individual bins were identified and are shown in Table 1 along with the algorithm giving the best combined overall test accuracy (64%) and PPV (82.9).

**Table 1:** Descriptive statistics, test accuracy, positive predictive values, significance, and relative cutoff for each bin (bins 1–9) and also for the combined algorithm

Bin number	Mass range (*m*/*z*)	Cutoff	Mann–Whitney *U* test	PPV (%)	Accuracy (%)
1	2200 to 2299	≥ 1.2	*p* = 0.0274	78.1	60.3
2	3500 to 3599	≥ 0.3	*p* = 0.1271	78.3	58.8
3	3600 to 3699	≥ 0.3	*p* = 0.1201	74.7	63.2
4	3800 to 3899	≥ 1.8	*p* = 0.0522	75.3	60.3
5	6500 to 6599	≤ 0.8	*p* = 0.1236	81.0	58.8
6	6600 to 6699	≤ 0.4	*p* = 0.0182	82.0	58.8
7	9100 to 9199	≤ 2.9	*p* = 0.0546	78.6	65.4
8	9200 to 9299	≤ 2.0	*p* = 0.0477	80.7	67.4
9	9300 to 9399	≤ 0.9	*p* = 0.0446	78.3	58.8
Combined	Algorithm	> 0.65	p = 0.0018	82.9	64.0

These nine bins (labeled bins 1 to 9) are also shown by arrows in Fig. 2 to indicate their relative positions within the spectra of median values for pregnant and negative outcomes. The nine bins selected include 100-*m*/*z* regions: 2200 to 2299, 3500 to 3599, 3600 to 3699, 3800 to 3899, 6500 to 6599, 6600 to 6699, 9100 to 9199, 9200 to 9299, and 9300 to 9399 and can be examined in Fig. 2.

Mass bin 1 and bin 6 performed well individually, each with *p* values less than 0.05 and PPV of 78.1% and 82%, respectively. However, in combination, the algorithm employing all nine bins produced a PPV of 82.9% with a *p* value of 0.0018 (Table 1); the box and whisker plot of the individual bins and the combined algorithm are also shown in Fig. 3. The descriptive statistics, test accuracy, positive predictive values, significance, and cutoff for each of these bins and also for the combined algorithm are shown in Table 1. The distributions of values for each of nine selected bins are also shown as box and whisker plots (Fig. 3).

## Discussion

Pregnancy rates from assisted reproduction are not improving despite the widespread adoption of morphokinetics and genetic testing [2]. The use of genomics, proteomics, and metabolomics to analyze embryo development, or embryo quality, are promising non-invasive alternatives, which may provide opportunities to improving pregnancy success in assisted reproduction [7]. However, in order to achieve this effectively, any new test needs to be quick and simple to appropriately fit into current embryology laboratory workflows.

Although, this study is based on a moderate and not thousands of cycles and samples; we believe that this is the largest database of its kind collected from a single center for the sole purpose of MALDI ToF analysis. This avoids the metabolic signature differences between IVF centers and peculiarities associated with the differences in culture media and embryologist practices.

Cortezzi et al. [17] examined embryo culture media by “metabolomic” analysis of smaller molecules (< 1500 *m*/*z*) resolving thousands of small ion signals. Complex mathematical modeling was required to fit data into one of two groups. One hundred percent of sensitivity and specificity are often being reported using decision tree analysis on the sample set. However, approaches like this invariably overfit the data and fail during testing with unknown, real-time samples. Complex omics-based approaches will undoubtedly lead to an explosion of information and a multitude of opportunities to examine embryos in culture and influence their development to result in optimal embryos. However, these methods are time-consuming and require skilled technicians and significant capital expenditure to operate successfully. Therefore, it is unlikely that many of these approaches will be able to influence embryo selection in a real-time clinical environment.

Here, a very simple probabilistic approach was adopted which is far less susceptible to overfitting. Unlike other approaches, MALDI ToF MS analysis of the culture media, which takes less than 20 min, could be a practical method to determine embryo quality and likelihood of pregnancy success, all during that critical window of time between blastocyst hatching and embryo transfer.

Recent developments in MALDI ToF mass spectrometer design are towards robust and reliable benchtop analyzers which are currently revolutionizing clinical microbiology testing [22, 23]. Furthermore, by applying a mass bin approach, it has been possible to identify and quantify individual protein profile differences [18, 19]. Many previous attempts to build effective MALDI ToF MS–based screening tests have failed because they have not rationalized the raw data into size bins. Multiple peaks are seen for any given molecule which is resolved by mass spectrometry and are largely due to isotopic variations. This becomes an exaggerated, and far more significant problem; the larger the complex molecules are, as the composition of carbon, oxygen, and nitrogen isotopes within a protein can vary so much that the variation in mass can span 10 to 100 s of daltons. This isotope variation will be a function of global locality but will also be predominantly a consequence of diet (and from foods that are grown all over the world). Thus, although high resolution is an analytical reality for mass spectrometry, resolving every possible peak to within 1 Da is actually a problem in certain clinical situations (when looking at large molecules) as it results in peak recognitions that are inconsistent and irrelevant to the clinical problem. Binning high-resolution mass data, to a size appropriate for the target molecules, like focusing, brings consistency to pattern recognition and relevant clinically information [24].

Therefore, given latest advances in instruments and data handling, if correctly focused, it is possible to create a MALDI ToF MS bench-side process to rapidly assess the potential viability of an embryo, without sampling the embryo itself.

In this study, we purposefully selected mass regions above 2000 *m*/*z* to avoid problems associated with measurement of small molecule metabolic variation. We examined a mass region (2000–17,000 *m*/*z*) to ensure the analysis of fluctuations in proteins and peptides which provide a better indication of the embryo’s functional biology and also fall within the optimal analytical performance of the MALDI ToF MS instrument. As such, the particular signatures of these molecules better indicate the quality of an embryo with respect to its likelihood of implanting and continuing as a viable pregnancy. In the complex analysis, this is not only an increase but also a decrease in biomolecules identified in the spectra (Fig. 2). Therefore, indicating that embryo viability is not necessarily about what is produced, but also what is being utilized. Thus, consumption of culture media components and secretion of metabolites and cytokines are tightly regulated. Monitoring this relations in combination (a spectral pattern) better reflect the complex molecular interactions which are taking place at this time in development, than single biomarker monitoring. Other proteomic approaches have attempted to identify and quantify specific proteins which are either raised or reduced in embryos and have developed a hierarchic classification of embryos on which to base selection [14]. This will limit the tests clinical efficacy.

The data presented here indicates that it would be possible to improve the selection of embryos for embryo transfer from which are more likely to develop into an ongoing pregnancy using MALDI ToF spectral analysis of spent embryo culture fluid .

In reality, 100% specificity is never going to be possible with an embryo selection test given that positive pregnancy is not only dependent on the quality of an embryo but also uterine receptivity during the implantation process. Implantation and the ability to maintain that implanted embryo are both complex and dynamic [25]. In this study, at least two of the women included in the analysis had recognizable and documented uterine problems (fibroids, adenomyosis, unicornate uterus), both of which repeatedly scored highly in our test but never achieved pregnancy.

## Conclusion

Despite much hype and expense, preimplantation genetic screening for chromosomal abnormalities has not improved the success rate of in vitro fertilization. Choosing which fertilized embryo to transfer to the womb and grading them on the basis of biological markers is the one remaining grand challenge in assisted reproduction.

We have developed a simple and direct method which takes less than 20 min to analyze spent culture fluid from blastocysts, at the point of embryo transfer, which can quickly identify embryos with the best chance of achieving ongoing pregnancy. Implementation of culture media analysis, using rapid and affordable methods like this, could form part of the standard preparation of embryos in culture prior to transfer, dramatically improve single embryo selection and ultimately increase the number of live births in assisted reproduction.
